# Bis(tri­ethyl­ammonium) chloranilate

**DOI:** 10.1107/S1600536813021405

**Published:** 2013-08-10

**Authors:** Kazuma Gotoh, Shinpei Maruyama, Hiroyuki Ishida

**Affiliations:** a Department of Chemistry, Faculty of Science, Okayama University, Okayama 700-8530, Japan

## Abstract

In the crystal structure of the title compound [systematic name: bis­(tri­ethyl­ammonium) 2,5-di­chloro-3,6-dioxo­cyclo­hexa-1,4-diene-1,4-diolate], 2C_6_H_16_N^+^·C_6_Cl_2_O_4_^2−^, the chloranilate anion lies on an inversion center. The tri­ethyl­ammonium cations are linked on both sides of the anion *via* bifurcated N—H⋯(O,O) and weak C—H⋯O hydrogen bonds to give a centrosymmetric 2:1 aggregate. The 2:1 aggregates are further linked by C—H⋯O hydrogen bonds into a zigzag chain running along [01-1].

## Related literature

For related structures, see: Dayananda *et al.* (2012 ▶); Gotoh *et al.* (2009 ▶, 2010 ▶); Yang (2007 ▶).
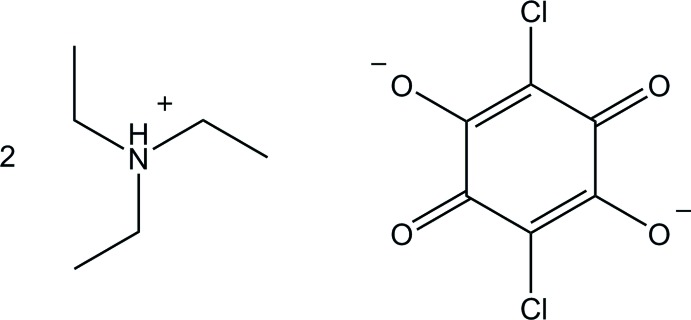


## Experimental

### 

#### Crystal data

2C_6_H_16_N^+^·C_6_Cl_2_O_4_^2−^
*M* *_r_* = 411.37
Triclinic, 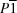

*a* = 7.7347 (5) Å
*b* = 8.5151 (8) Å
*c* = 9.3913 (7) Å
α = 64.388 (4)°
β = 68.435 (3)°
γ = 79.060 (5)°
*V* = 518.36 (7) Å^3^
*Z* = 1
Mo *K*α radiation
μ = 0.34 mm^−1^
*T* = 180 K
0.65 × 0.31 × 0.21 mm

#### Data collection

Rigaku R-AXIS RAPID II diffractometer
Absorption correction: numerical (*NUMABS*; Higashi, 1999 ▶) *T*_min_ = 0.858, *T*_max_ = 0.932
10264 measured reflections
3012 independent reflections
2561 reflections with *I* > 2σ(*I*)
*R*_int_ = 0.078

#### Refinement

*R*[*F* ^2^ > 2σ(*F* ^2^)] = 0.044
*wR*(*F* ^2^) = 0.121
*S* = 1.06
3012 reflections
125 parameters
H atoms treated by a mixture of independent and constrained refinement
Δρ_max_ = 0.48 e Å^−3^
Δρ_min_ = −0.26 e Å^−3^

### 

Data collection: *PROCESS-AUTO* (Rigaku/MSC, 2004 ▶); cell refinement: *PROCESS-AUTO*; data reduction: *CrystalStructure* (Rigaku/MSC, 2004 ▶); program(s) used to solve structure: *SHELXS97* (Sheldrick, 2008 ▶); program(s) used to refine structure: *SHELXL97* (Sheldrick, 2008 ▶); molecular graphics: *ORTEP-3 for Windows* (Farrugia, 2012 ▶); software used to prepare material for publication: *CrystalStructure* (Rigaku/MSC, 2004 ▶) and *PLATON* (Spek, 2009 ▶).

## Supplementary Material

Crystal structure: contains datablock(s) General, I. DOI: 10.1107/S1600536813021405/lh5635sup1.cif

Structure factors: contains datablock(s) I. DOI: 10.1107/S1600536813021405/lh5635Isup2.hkl

Supplementary material file. DOI: 10.1107/S1600536813021405/lh5635Isup3.cml

Additional supplementary materials:  crystallographic information; 3D view; checkCIF report

## Figures and Tables

**Table 1:** Hydrogen-bond geometry (Å, °)

*D*—H⋯*A*	*D*—H	H⋯*A*	*D*⋯*A*	*D*—H⋯*A*
N1—H1⋯O1	0.867 (18)	1.942 (18)	2.7601 (15)	156.9 (15)
N1—H1⋯O2^i^	0.867 (18)	2.339 (17)	2.9377 (14)	126.4 (14)
C5—H5*C*⋯O1	0.98	2.57	3.2911 (19)	131
C9—H9*B*⋯O1^ii^	0.98	2.55	3.5315 (17)	177

Symmetry codes: (i) 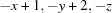; (ii) 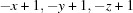.

**Fig. 1.**
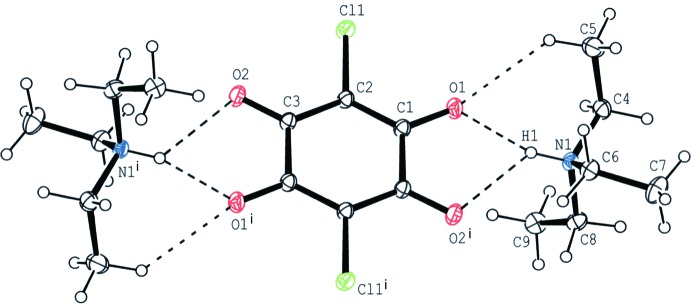
The molecular structure of the title compound, with the atom-labeling. Displacement ellipsoids of non-H atoms are drawn at the 30% probability level. The dashed lines indicate N—H···O and C—H···O hydrogen bonds. [Symmetry code: (i) -x + 1, -y + 2, -z.]

**Fig. 2.**
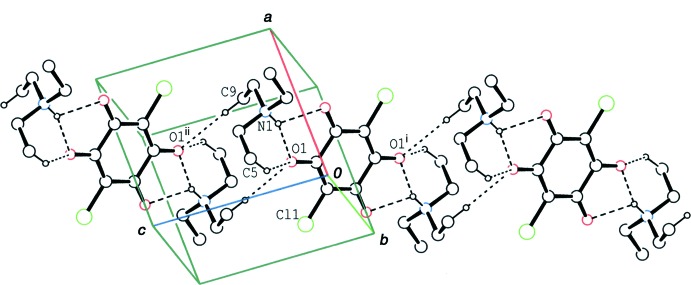
A partial packing diagram of the title compound. The dashed lines indicate N—H···O and C—H···O hydrogen bonds. H atoms of the ethyl groups not involved in the C—H···O hydrogen bonds have been omitted. [Symmetry codes: (i) -x + 1, -y + 2, -z; (ii) -x + 1, -y + 1, -z.]

## supplementary crystallographic information

### 1. Comment

The title compound was prepared in order to extend our study on *D*—H···*A* hydrogen bonding (*D* = N, O, or C; *A* = N, O or Cl) in amine–chloranilic acid systems (Gotoh *et al.*, 2009, 2010). For the tertiary amine–chloranilic acid systems, crystal structures of bis(hexamethylenetetraminium) chloranilate tetrahydrate (Yang, 2007), triethylammonium hydrogen chloranilate (Gotoh *et al.*, 2010) and triprolidinium dichloranilate–chloranilic acid–methanol–water (2/1/2/2) (Dayananda *et al.*, 2012) have been reported.

In the crystal structure of the title compound, an acid-base interaction involving proton transfer is observed between chloranilic acid and triethylamine, and one chloranilate anion and two triethylammnoium cations are linked by bifurcated N—H···O and weak C—H···O hydrogen bonds (Table 1) to afford a centrosymmetric 2:1 aggregate (Fig. 1). The 2:1 aggregates are further linked by intermolecular C—H···O hydrogen bonds, forming a zigzag chain running along the [011] direction (Fig. 2).

### 2. Experimental

Single crystals were obtained by slow evaporation from an acetonitrile solution (100 ml) of chloranilic acid (100 mg) and triethylamine (97 mg) at room temperature.

### 3. Refinement

C-bound H atoms were positioned geometrically (C—H = 0.98 or 0.99 Å) and refined as riding, allowing for free rotation of the methyl group. *U*_iso_(H) values were set at 1.2*U*_eq_(C) or 1.5*U*_eq_(methyl C). The N-bound H atom was found in a difference Fourier map and refined isotropically. The refined N—H distance is 0.867 (18) Å.

### Crystal data

**Table:**

2C_6_H_16_N^+^·C_6_Cl_2_O_4_^2^^−^	*Z* = 1
*M**_r_* = 411.37	*F*(000) = 220.00
Triclinic, *P*1	*D*_x_ = 1.318 Mg m^−^^3^
Hall symbol: -P 1	Mo *K*α radiation, λ = 0.71075 Å
*a* = 7.7347 (5) Å	Cell parameters from 8559 reflections
*b* = 8.5151 (8) Å	θ = 3.1–30.1°
*c* = 9.3913 (7) Å	µ = 0.34 mm^−^^1^
α = 64.388 (4)°	*T* = 180 K
β = 68.435 (3)°	Block, brown
γ = 79.060 (5)°	0.65 × 0.31 × 0.21 mm
*V* = 518.36 (7) Å^3^	

### Data collection

**Table:**

Rigaku R-AXIS RAPID II diffractometer	2561 reflections with *I* > 2σ(*I*)
Detector resolution: 10.00 pixels mm^-1^	*R*_int_ = 0.078
ω scans	θ_max_ = 30.0°, θ_min_ = 3.1°
Absorption correction: numerical (*NUMABS*; Higashi, 1999)	*h* = −10→10
*T*_min_ = 0.858, *T*_max_ = 0.932	*k* = −11→11
10264 measured reflections	*l* = −13→13
3012 independent reflections	

### Refinement

**Table:**

Refinement on *F*^2^	Primary atom site location: structure-invariant direct methods
Least-squares matrix: full	Secondary atom site location: difference Fourier map
*R*[*F*^2^ > 2σ(*F*^2^)] = 0.044	Hydrogen site location: inferred from neighbouring sites
*wR*(*F*^2^) = 0.121	H atoms treated by a mixture of independent and constrained refinement
*S* = 1.06	*w* = 1/[σ^2^(*F*_o_^2^) + (0.0654*P*)^2^] where *P* = (*F*_o_^2^ + 2*F*_c_^2^)/3
3012 reflections	(Δ/σ)_max_ = 0.001
125 parameters	Δρ_max_ = 0.48 e Å^−^^3^
0 restraints	Δρ_min_ = −0.26 e Å^−^^3^

### Special details

**Table:**

Geometry. All e.s.d.'s (except the e.s.d. in the dihedral angle between two l.s. planes) are estimated using the full covariance matrix. The cell e.s.d.'s are taken into account individually in the estimation of e.s.d.'s in distances, angles and torsion angles; correlations between e.s.d.'s in cell parameters are only used when they are defined by crystal symmetry. An approximate (isotropic) treatment of cell e.s.d.'s is used for estimating e.s.d.'s involving l.s. planes.
Refinement. Refinement of *F*^2^ against ALL reflections. The weighted *R*-factor *wR* and goodness of fit *S* are based on *F*^2^, conventional *R*-factors *R* are based on *F*, with *F* set to zero for negative *F*^2^. The threshold expression of *F*^2^ > σ(*F*^2^) is used only for calculating *R*-factors(gt) *etc*. and is not relevant to the choice of reflections for refinement. *R*-factors based on *F*^2^ are statistically about twice as large as those based on *F*, and *R*- factors based on ALL data will be even larger.

### Fractional atomic coordinates and isotropic or equivalent isotropic displacement parameters (Å^2^)

**Table:**

	*x*	*y*	*z*	*U*_iso_*/*U*_eq_	
Cl1	0.14870 (4)	0.93266 (4)	0.32667 (3)	0.03021 (13)	
O1	0.44257 (12)	0.67475 (10)	0.24001 (11)	0.0322 (2)	
O2	0.26077 (12)	1.26690 (10)	0.03171 (10)	0.0326 (2)	
N1	0.67048 (13)	0.38363 (13)	0.23709 (12)	0.0242 (2)	
C1	0.46352 (15)	0.82853 (14)	0.13459 (13)	0.0237 (2)	
C2	0.34397 (15)	0.96966 (14)	0.14976 (13)	0.0235 (2)	
C3	0.36699 (15)	1.13964 (14)	0.02453 (14)	0.0235 (2)	
C4	0.54491 (18)	0.26759 (16)	0.40054 (15)	0.0313 (3)	
H4A	0.5321	0.3103	0.4868	0.038*	
H4B	0.6024	0.1485	0.4333	0.038*	
C5	0.35317 (19)	0.25958 (19)	0.39536 (18)	0.0396 (3)	
H5A	0.2712	0.1966	0.5086	0.059*	
H5B	0.3625	0.1988	0.3247	0.059*	
H5C	0.3018	0.3782	0.3494	0.059*	
C6	0.69491 (17)	0.33514 (16)	0.09491 (15)	0.0297 (3)	
H6A	0.7649	0.4268	−0.0105	0.036*	
H6B	0.5708	0.3319	0.0884	0.036*	
C7	0.7960 (2)	0.1614 (2)	0.1098 (2)	0.0435 (4)	
H7A	0.8200	0.1442	0.0075	0.065*	
H7B	0.7191	0.0679	0.2051	0.065*	
H7C	0.9143	0.1595	0.1265	0.065*	
C8	0.85542 (16)	0.39642 (17)	0.24970 (15)	0.0295 (3)	
H8A	0.9018	0.2780	0.3091	0.035*	
H8B	0.9459	0.4451	0.1360	0.035*	
C9	0.84408 (19)	0.50912 (17)	0.34006 (17)	0.0342 (3)	
H9A	0.9693	0.5226	0.3355	0.051*	
H9B	0.7662	0.4542	0.4567	0.051*	
H9C	0.7894	0.6239	0.2868	0.051*	
H1	0.618 (2)	0.487 (2)	0.209 (2)	0.034 (4)*	

### Atomic displacement parameters (Å^2^)

**Table:**

	*U*^11^	*U*^22^	*U*^33^	*U*^12^	*U*^13^	*U*^23^
Cl1	0.02658 (19)	0.03015 (19)	0.02264 (18)	0.00392 (12)	−0.00412 (13)	−0.00561 (13)
O1	0.0340 (5)	0.0216 (4)	0.0265 (4)	0.0031 (3)	−0.0046 (4)	−0.0025 (3)
O2	0.0326 (5)	0.0247 (4)	0.0290 (5)	0.0080 (3)	−0.0055 (4)	−0.0078 (4)
N1	0.0252 (5)	0.0216 (5)	0.0220 (4)	0.0037 (3)	−0.0086 (4)	−0.0062 (4)
C1	0.0254 (5)	0.0227 (5)	0.0216 (5)	0.0019 (4)	−0.0092 (4)	−0.0072 (4)
C2	0.0235 (5)	0.0233 (5)	0.0196 (5)	0.0023 (4)	−0.0067 (4)	−0.0063 (4)
C3	0.0246 (5)	0.0219 (5)	0.0230 (5)	0.0030 (4)	−0.0094 (4)	−0.0082 (4)
C4	0.0327 (6)	0.0267 (6)	0.0254 (6)	0.0000 (4)	−0.0062 (5)	−0.0054 (5)
C5	0.0352 (7)	0.0392 (7)	0.0414 (7)	−0.0081 (5)	−0.0058 (6)	−0.0159 (6)
C6	0.0326 (6)	0.0317 (6)	0.0267 (6)	0.0028 (5)	−0.0139 (5)	−0.0114 (5)
C7	0.0479 (8)	0.0454 (8)	0.0561 (9)	0.0166 (6)	−0.0296 (7)	−0.0339 (7)
C8	0.0262 (6)	0.0355 (6)	0.0272 (6)	0.0026 (4)	−0.0106 (5)	−0.0128 (5)
C9	0.0355 (7)	0.0370 (7)	0.0330 (6)	−0.0009 (5)	−0.0120 (5)	−0.0159 (6)

### Geometric parameters (Å, º)

**Table:**

Cl1—C2	1.7390 (11)	C5—H5B	0.9800
O1—C1	1.2502 (13)	C5—H5C	0.9800
O2—C3	1.2403 (13)	C6—C7	1.5103 (18)
N1—C4	1.4943 (15)	C6—H6A	0.9900
N1—C6	1.5002 (15)	C6—H6B	0.9900
N1—C8	1.5056 (15)	C7—H7A	0.9800
N1—H1	0.867 (18)	C7—H7B	0.9800
C1—C2	1.3960 (15)	C7—H7C	0.9800
C1—C3^i^	1.5394 (16)	C8—C9	1.5054 (17)
C2—C3	1.4079 (15)	C8—H8A	0.9900
C3—C1^i^	1.5394 (16)	C8—H8B	0.9900
C4—C5	1.517 (2)	C9—H9A	0.9800
C4—H4A	0.9900	C9—H9B	0.9800
C4—H4B	0.9900	C9—H9C	0.9800
C5—H5A	0.9800		
			
C4—N1—C6	113.81 (10)	H5B—C5—H5C	109.5
C4—N1—C8	111.29 (9)	N1—C6—C7	113.74 (10)
C6—N1—C8	111.25 (9)	N1—C6—H6A	108.8
C4—N1—H1	108.1 (10)	C7—C6—H6A	108.8
C6—N1—H1	103.7 (10)	N1—C6—H6B	108.8
C8—N1—H1	108.2 (10)	C7—C6—H6B	108.8
O1—C1—C2	125.01 (10)	H6A—C6—H6B	107.7
O1—C1—C3^i^	116.31 (9)	C6—C7—H7A	109.5
C2—C1—C3^i^	118.67 (9)	C6—C7—H7B	109.5
C1—C2—C3	123.37 (10)	H7A—C7—H7B	109.5
C1—C2—Cl1	118.69 (8)	C6—C7—H7C	109.5
C3—C2—Cl1	117.83 (8)	H7A—C7—H7C	109.5
O2—C3—C2	125.12 (10)	H7B—C7—H7C	109.5
O2—C3—C1^i^	116.95 (9)	C9—C8—N1	112.57 (10)
C2—C3—C1^i^	117.93 (9)	C9—C8—H8A	109.1
N1—C4—C5	112.84 (11)	N1—C8—H8A	109.1
N1—C4—H4A	109.0	C9—C8—H8B	109.1
C5—C4—H4A	109.0	N1—C8—H8B	109.1
N1—C4—H4B	109.0	H8A—C8—H8B	107.8
C5—C4—H4B	109.0	C8—C9—H9A	109.5
H4A—C4—H4B	107.8	C8—C9—H9B	109.5
C4—C5—H5A	109.5	H9A—C9—H9B	109.5
C4—C5—H5B	109.5	C8—C9—H9C	109.5
H5A—C5—H5B	109.5	H9A—C9—H9C	109.5
C4—C5—H5C	109.5	H9B—C9—H9C	109.5
H5A—C5—H5C	109.5		
			
O1—C1—C2—C3	−176.68 (11)	Cl1—C2—C3—C1^i^	−178.12 (8)
C3^i^—C1—C2—C3	1.97 (19)	C6—N1—C4—C5	−56.62 (13)
O1—C1—C2—Cl1	−0.54 (17)	C8—N1—C4—C5	176.71 (10)
C3^i^—C1—C2—Cl1	178.11 (8)	C4—N1—C6—C7	−66.29 (14)
C1—C2—C3—O2	177.84 (11)	C8—N1—C6—C7	60.39 (14)
Cl1—C2—C3—O2	1.67 (17)	C4—N1—C8—C9	−74.58 (13)
C1—C2—C3—C1^i^	−1.95 (18)	C6—N1—C8—C9	157.36 (10)

Symmetry code: (i) −*x*+1, −*y*+2, −*z*.

### Hydrogen-bond geometry (Å, º)

**Table:**

*D*—H···*A*	*D*—H	H···*A*	*D*···*A*	*D*—H···*A*
N1—H1···O1	0.867 (18)	1.942 (18)	2.7601 (15)	156.9 (15)
N1—H1···O2^i^	0.867 (18)	2.339 (17)	2.9377 (14)	126.4 (14)
C5—H5*C*···O1	0.98	2.57	3.2911 (19)	131
C9—H9*B*···O1^ii^	0.98	2.55	3.5315 (17)	177

Symmetry codes: (i) −*x*+1, −*y*+2, −*z*; (ii) −*x*+1, −*y*+1, −*z*+1.
